# Cryptic diversity in southern African kelp

**DOI:** 10.1038/s41598-024-61336-4

**Published:** 2024-05-14

**Authors:** Pedro Madeira, Maggie M. Reddy, Jorge Assis, John J. Bolton, Mark D. Rothman, Robert J. Anderson, Lineekela Kandjengo, Anja Kreiner, Melinda A. Coleman, Thomas Wernberg, Olivier De Clerck, Frederik Leliaert, Salomão Bandeira, Abdul M. Ada, João Neiva, Gareth A. Pearson, Ester A. Serrão

**Affiliations:** 1 CCMAR, University of Algarve, Gambelas, Faro, Portugal; 2 Department of Biological Sciences, University of Cape Town, Cape Town, 7701 South Africa; 3 Department of Environment, Forestry and Fisheries, Private Bag X2, Vlaeberg, 8012 South Africa; 4 Department of Fisheries and Ocean Sciences, University of Namibia, Sam Nujoma Campus, Henties Bay, Namibia; 5 National Marine Information and Research Centre, Ministry of Fisheries and Marine Resources, Swakopmund, Namibia; 6 New South Wales Fisheries, National Marine Science Centre, 2 Bay Drive, Coffs Harbour, NSW 2450 Australia; 7 National Marine Science Centre, Southern Cross University, 2 Bay Drive, Coffs Harbour, NSW 2450 Australia; 8 UWA Oceans Institute and School of Biological Sciences, University of Western Australia, 35 Stirling Highway, Crawley, WA 6009 Australia; 9 Biology Department, Ghent University, Krijgslaan 281 S8, 9000 Ghent, Belgium; 10 Meise Botanic Garden, 1860 Meise, Belgium; 11 Department of Biological Sciences, Eduardo Mondlane University, Maputo, Mozambique; 12 Department of Biological Sciences, Louisiana State University, Baton Rouge, LA 70803 USA; 13 Faculty of Bioscience and Aquaculture, Nord Universitet, Bodø, Norway

**Keywords:** Population genetics, Genetic hybridization, Marine biology

## Abstract

The southern coast of Africa is one of the few places in the world where water temperatures are predicted to cool in the future. This endemism-rich coastline is home to two sister species of kelps of the genus *Ecklonia maxima* and *Ecklonia radiata*, each associated with specific thermal niches, and occuring primarily on opposite sides of the southern tip of Africa. Historical distribution records indicate that *E. maxima* has recently shifted its distribution ~ 70 km eastward, to sites where only *E. radiata* was previously reported. The contact of sister species with contrasting thermal affinities and the occurrence of mixed morphologies raised the hypothesis that hybridization might be occurring in this contact zone. Here we describe the genetic structure of the genus *Ecklonia* along the southern coast of Africa and investigate potential hybridization and cryptic diversity using a combination of nuclear microsatellites and mitochondrial markers. We found that both species have geographically discrete genetic clusters, consistent with expected phylogeographic breaks along this coastline. In addition, depth-isolated populations were found to harbor unique genetic diversity, including a third *Ecklonia* lineage. Mito-nuclear discordance and high genetic divergence in the contact zones suggest multiple hybridization events between *Ecklonia* species. Discordance between morphological and molecular identification suggests the potential influence of abiotic factors leading to convergent phenotypes in the contact zones. Our results highlight an example of cryptic diversity and hybridization driven by contact between two closely related keystone species with contrasting thermal affinities.

## Introduction

Climate-driven ocean contact zones, caused by range shifts of reproductively compatible but differentiated genetic lineages, provide evolutionary opportunities by promoting rapid genome recombination and novel selective variants^1,2^. Species can undergo rapid divergence when isolated during climate extremes and subsequent secondary contact can result in outcomes ranging from complete panmixia to reproductive isolation^3–6^.

The genomic consequences of contact zones may be especially relevant for species with restricted dispersal abilities. In marine environments, currents can shape and restrict dispersal, causing limited connectivity that favours evolutionary processes at local scales, giving isolated populations time to diverge. This is the case for kelps, which form massive underwater forests across many temperate and Arctic coastal regions^7^, but have been undergoing distributional range shifts and contractions as ocean temperatures change^8–11^. While range shifts can result in the loss of populations and total kelp biomass^7,12,13^, they may also be associated with an increase in evolutionary potential when contact zones between different lineages of the same, or closely related species, are created. For example, hybridization may accelerate local adaptation of seaweed to previously unsuitable conditions^14,15^. Several examples of kelp hybridization have been described either experimentally^16–19^ or in the field^20–24^. In changing oceans, it is important to understand how shifts in kelp distribution may result in niche overlap between closely related species, putatively resulting in admixture between genetically distinct populations and species. Given the morphological plasticity of many kelps^25–28^, molecular data are essential for distinguishing between cryptic populations with atypical morphologies and hybrids as well as for the identification of populations with unique genetic diversity.

Climate-driven distributional changes have been observed within the genus *Ecklonia* (*E. maxima* and *E. radiata*) along the coast of southern Africa. The range of *Ecklonia maxima* has shifted eastward^8^, while the loss of a unique northern hemisphere population of *E. radiata* was recently reported from Oman, potentially due to the impact of climate change^12^. *Ecklonia radiata* is among the most dominant kelps in the southern hemisphere, with a warm-temperate distribution ranging from New Zealand and Australia to the south and eastern coasts of southern Africa^12,29^ (upper thermal tolerance range 21.2–26.5 °C^29^). *Ecklonia radiata* is generally small (40 cm up to 2 m long), occurring typically in the intertidal to subtidal depths of 30 m. Rarer and deeper populations have also been recorded at depths reaching 80 m, under suitable conditions^30,31^. It forms extensive beds in Australasia and usually sparser populations in sheltered subtidal and intertidal regions in most of its South African distribution^8,12,29^. In Africa, *E. radiata* extends eastwards from Cape Agulhas to southern Mozambique^12^ in addition to a single population in the Cape Peninsula^21^. There are also records of previously undescribed deep populations in northeastern South Africa and Mozambique and past populations were recorded in similar habitats as far north as Oman^12^. A second species, *Ecklonia maxima* is abundant on the west coast of South Africa in the cool-temperate Benguela region. *Ecklonia maxima* also occurs in the biogeographical overlap region between the west coast and warm temperature south coast and evidence suggests that gametophytes and small sporophytes are capable of surviving in nutrient-replete cultures at temperatures up to 22 °C^16^. It forms large marine forests, often growing to several meters in length, has a long, hollow stipe (maximum 15 m^32^), with a float at the top holding the fronds^32^. It is the main canopy-forming kelp in < 6 m depth in this region, although it can be found down to 30 m under certain conditions^33^. Its northern limit is in southern Namibia (Anderson et al.^34^, pers obs MMR) and it has recently expanded ~ 70 km eastwards around the southern tip of South Africa, reaching the De Hoop Nature Reserve, ca. 2006^8^.

The two species can be found in contact zones along the South African coast. Previous genetic studies have shown that *E. radiata* and *E. maxima* are sister species that form a clade separate from the east Asian *Ecklonia*^21,25,35^. Interestingly, the taxonomic affinity of *E. radiata* from the Cape Peninsula was unclear (based on nuclear ITS and organellar markers), with individuals presenting atypical morphologies, generally *E. maxima*-like, but with *E. radiata*-like rugose and serrated fronds, solid stipes, and with discordant nuclear and mitochondrial gene trees, suggesting a potential hybrid origin^21^. *Ecklonia radiata* and *E. maxima* are able to produce viable hybrid sporophytes in laboratory experiments^16^ raising the question of whether similar events occur in nature. Since both species can display morphological plasticity that makes morphology-based identification difficult, particularly for *E. radiata*^8,21,29^, molecular data from multiple markers are essential to identify lineages.

This study aims to understand the impact of niche overlap on the genetic diversity of the two *Ecklonia* species and investigate possible hybridization events. To achieve this, we sampled the entire known distribution of *Ecklonia* in southern Africa, including some of the northernmost localities of the known distribution for both species, the southernmost point, as well as deep offshore populations from the south Atlantic and southern South Africa. Nuclear and mitochondrial markers were compared to identify possible introgression in contact zones and unique genetic diversity in edge and deep populations. In addition, we modelled the distribution of both species to evaluate niche differentiation and the probability of range overlap. Our analyses reveal marked geographic patterns of genetic structure in both *Ecklonia* species and indicate that the deep populations of *Ecklonia* in southern Africa are important refugia of unique genetic diversity. Our findings also provide evidence of hybridization in the contact zones between the two species and raise the hypothesis that hybrids are more likely to be adapted to the broader niche of *E. radiata*.

## Material and methods

### Sampling, DNA extraction, genotyping and sequencing

**Figure 1:**
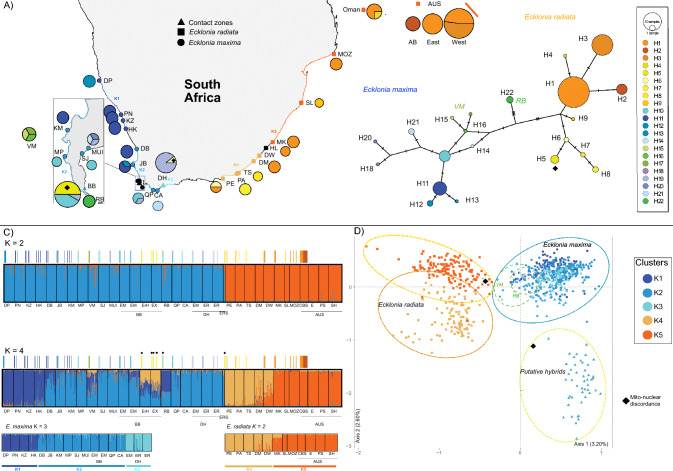
Sampling locations of *Ecklonia* in southern Africa and genetic structure inferred from mtDNA and microsatellite genotypes. Warm colors (orange–yellow) identify *E. radiata* haplogroups and clusters while cold colors (blue–purple) identify *E. maxima*. Colored bars above STRUCTURE plots indicate individuals’ haplotype. Black diamond marks denote individuals for which there was a discordant species attribution between mitochondrial and nuclear markers. A) Geographic distribution of COX1 haplotypes (pie charts) and STRUCTURE clusters (borders) for K = 8 and B) haplotype network showing haplotype genealogy. C) Structure plots assuming K = 2 (top) and K = 4 (middle) and species specific (bottom) genetic clusters and D) FCA scatter plot based on the clusters defined K = 4, circles colored according to mtDNA haplotypes present in the cluster. Map was created using a custom R (https://cran.r-project.org/) script on version 4.3.0 and edited using Affinity Designer, version 2.3.1.

Populations of *Ecklonia* were sampled from shallow and deeper reefs spanning the entire known distribution of the genus off the coast of southern Africa and Vema Seamount (roughly 1000 km offshore from the west coast of South Africa; closest to Doringbaai: DB on Fig. 1). Further sampling effort was focused on the two identified contact zones (BB, DH) and on deeper populations (VM, RB), where individuals with atypical morphologies were identified. These samples were identified as either *E. maxima* (EMDH) or *E. radiata* (ERHOO, ERDH, ERSDH) (see Table 1 for sample site abbreviations) in the field, based on morphological traits considered to be diagnostic, such as hollow stipe for *E. maxima* or spiny and serrated blades for *E. radiata*. Individuals with atypical morphologies were also collected such as smooth bladed *E. radiata* (ERHOO, ERSDH) and plants similar to those described by Rothman et al.^21^ as “*E. cf. radiata/maxima*” (ERhBOT, EXBB), a putative hybrid. Additionally, four populations of *E. radiata* were included from shallow waters off the coast of New South Wales, Australia, for comparison with South African samples and to provide a better representation of the species’ genetic diversity (Table 1).

**Table 1:** Sampling events of *Ecklonia* populations in southern Africa and Australia, ordered east to west.

#	Region	Species	Site	Lat.	Lon.	N	A	Â	PÂ	H_E_	H_O_	F_IS_
1	Diaz Point (Namibia)	EM	DP	− 26.643683	15.088839	24	2.750	2.64 ± 0.12	1.93 ± 0.47	0.238	0.108	0.551*
2	Port Nolloth	EM	PN	− 29.273186	16.883044	24	3.250	3.1 ± 0.13	0.00 ± 0	0.396	0.341	0.143*
3	Kleinzee	EM	KZ	− 29.707946	17.054532	24	3.375	3.22 ± 0.14	2.75 ± 0.96	0.424	0.383	0.099
4	Hondeklipbaai	EM	HK	− 30.328581	17.296195	24	4.000	3.82 ± 0.18	0.13 ± 0.34	0.487	0.370	0.246*
5	Doringbaai	EM	DB	− 31.821056	18.239235	24	4.000	3.82 ± 0.13	0.84 ± 0.37	0.351	0.323	0.082
6	Jacobs Bay	EM	JB	− 32,968,981	17.89097	24	7.250	6.86 ± 0.23	1.92 ± 0.56	0.657	0.581	0.118*
7	Kommetjie	EM	KM	− 34.140312	18.329195	24	8.875	8.34 ± 0.2	0.33 ± 0.53	0.642	0.531	0.176*
8	Millers Point	EM	MP	− 34.23333	18.475000	24	7.250	6.76 ± 0.22	4.21 ± 0.95	0.583	0.561	0.038
9	Vema Seamount (South Atlantic)	EM	VM	− 31.633333	8.333333	24	5.00	4.71 ± 0.17	1.84 ± 0.63	0.393	0.374	0.051
10	St. James False Bay	EM	SJ	− 34.117724	18.460839	24	7.500	6.98 ± 0.26	3.50 ± 0.93	0.600	0.583	0.029
11	Muizenberg	EM	MUI	− 34.174831	18.475133	24	7.875	7.37 ± 0.24	1.00 ± 0.52	0.636	0.547	0.142*
12	Buffels Bay/Bortjiesrif	EM	BB	− 34.320198	18.461701	24	7.125	6.71 ± 0.17	1.20 ± 0.67	0.532	0.474	0.111*
13	Buffels Bay/Bortjiesrif	EM	BB	− 34.322097	18.4660387	24	6.750	6.2 ± 0.24	1.75 ± 0.67	0.559	0.454	0.191*
14	Buffels Bay/Bortjiesrif	ERh	BB	− 34.313772	18.465945	24	4.625	4.3 ± 0.15	0.00 ± 0.03	0.375	0.326	0.133*
15	Buffels Bay/Bortjiesrif	EX	BB	− 34.320198	18.461701	24	5.125	4.72 ± 0.27	1.87 ± 0.64	0.415	0.385	0.073
16	Rocky Bank	Esp	RB	− 34.59444	18.72222	22	3.000	2.93 ± 0.08	0.05 ± 0.22	0.313	0.301	0.041
17	Quoin Point	EM	QP	− 34.785546	19.6458134	24	7.500	7.21 ± 0.15	0.98 ± 0.39	0.602	0.483	0.202*
18	Cape Agulhas	EM	CA	− 34.823375	20.02231	24	6.500	6.18 ± 0.16	0.18 ± 0.39	0.636	0.629	0.012
19	De Hoop, Koppie Alleen	EM	DH	− 34.478188	20.511482	24	3.875	3.72 ± 0.13	0.00 ± 0	0.443	0.397	0.108*
20	De Hoop, Koppie Alleen	ERS	DH	− 34.4835082	20.5358989	24	4.625	4.44 ± 0.14	0.03 ± 0.17	0.530	0.479	0.097*
21	De Hoop, Koppie Alleen	ER	DH	− 34.478188	20.511482	5	3.625	3.54 ± 0.09	0.00 ± 0	0.488	0.464	0.052
22	De Hoop, Koppie Alleen	ER	DH	− 34.478188	20.511482	22	3.625	3.61 ± 0.09	0.18 ± 0.38	0.687	0.544	0.229*
23	Port Elizabeth	ER	PE	− 33.984444	25.670555	22	2.375	2.34 ± 0.06	1.00 ± 0.03	0.218	0.176	0.197*
24	Port Alfred	ER	PA	− 33.613951	26.889529	24	2.875	2.83 ± 0.09	0.00 ± 0	0.268	0.241	0.104
25	Three Sisters	ER	TS	− 33.558586	27.030783	20	3.875	3.7 ± 0.15	0.00 ± 0	0.362	0.332	0.086
26	Double Mouth	ER	DM	− 32.724734	28.315403	24	4.500	4.5 ± 0	1.04 ± 0.2	0.551	0.500	0.096*
27	Dwesa	ER	DW	− 32.30736	28.830793	24	6.000	5.72 ± 0.15	2.31 ± 0.71	0.542	0.504	0.071
28	Hluleka	ER	HL	− 31.821023	29.314165	2	–	–	–	–	–	–
29	Mkhambathi	ER	MK	− 31.317426	29.972852	23	2.625	2.58 ± 0.07	0.02 ± 0.15	0.395	0.406	− 0.029
30	St. Lucia	ER	SL	− 28.378889	32.442261	11	2.875	2.76 ± 0.14	0.00 ± 0.01	0.323	0.322	0.005
31	Zavora (Mozambique)	ER	MOZ	− 24.444444	35.35889	24	3.375	3.27 ± 0.12	0.20 ± 0.4	0.379	0.291	0.237*
32	Charlsworth Bay (AUS)	ER	CBS	− 30.267606	153.143319	19	4.875	4.1 ± 0.3	3.01 ± 1.05	0.384	0.273	0.293*
33	Eden (AUS)	ER	E	− 37.072117	149.907472	24	6.125	5.62 ± 0.26	5.31 ± 1.47	0.463	0.382	0.178*
34	Port Stephens (AUS)	ER	PS	− 32.717747	152.141544	23	5.250	4.96 ± 0.18	3.24 ± 0.63	0.443	0.315	0.292*
35	Shellharbour (AUS)	ER	SH	− 34.592694	150.880494	29	6.250	5.28 ± 0.46	3.35 ± 1.28	0.408	0.349	0.147*

All sites are in South Africa unless otherwise indicated. In situ species morphological identification is shown. EM: *E. maxima*, ER: *E. radiata*, ERh/EX: putative hybrids, Esp: Unidentified *Ecklonia* Genetic diversity measured using the 8 microsatellite loci is reported for each sampling event. A: mean allelic richness; Â: normalized allelic richness (for N = 20); PÂ: normalized number of private alleles (for N = 20); H_E_: Nei’s gene diversity; H_O_: observed heterozygosity; F_IS_: inbreeding coefficient (*indicates significantly different from zero at *p* < 0.05).

Silica-dried tissue from South African samples was macerated with 3 mm tungsten beads in a Tissue lyser II (Qiagen, Hilden, Germany) for 3 min. Genomic DNA was extracted using the NucleoSpin® 96 Tissue kit (Macherey–Nagel, Duren, Germany), following an adapted version of the supplier’s protocol. Extracted DNA was diluted 1:100 before PCR amplification. DNA from Australian samples was extracted by grinding 50 mg of frozen tissue, followed by an extraction and cleaning of total genomic DNA using the DNeasy plant DNA & Pro clean up kit (Qiagen). A total of 30 previously described microsatellite primers were tested, 12 *E. cava* primers^36^, 10 *E. radiata* primers^37^ and 8 *E. radicosa* primers^38^. Of these, 8 were polymorphic with consistent amplification in both *E. maxima* and *E. radiata* and were used to produce a multi-locus genotype matrix for all samples (Primer sequences and amplification are detailed in Table S1). After amplification, fragment size was analyzed on an ABI PRISM3130xl (Applied Biosystems) with GeneScan Liz 500 size standard (Applied Biosystems). Allele sizes were manually scored using STRAND (https://vgl.ucdavis.edu/STRand), binned with the R (R Core Team, 2023) package MsatAllele^39^ and manually reviewed to resolve ambiguities.

Complementary COX1 sequences for selected samples were obtained using the GAZF2 and GAZR2 primers previously published by^40^ (primer sequences and PCR conditions are detailed in Table S1). Sequencing was performed on an ABI PRISM3130xl (Applied Biosystems) automated sequencer at CCMAR. A total of 77 sequences were generated for southern African *Ecklonia*; 45 *E. maxima* sequences from 14 sites in South Africa and 3 from Namibia, 26 *E. radiata* sequences from 9 sites in South Africa and 3 from Mozambique, and 6 *Ecklonia spp.* sequences from 2 deep populations from Vema Seamount (n = 3) and Rocky Bank (n = 3) (Supplementary Table S2). Additionally, 128 *E. radiata* sequences from Australia and 4 from Oman were obtained from GenBank and added to the dataset (Supplementary Table S2).

### Genetic analyses

Genetic diversity per site for the nuclear markers was estimated with allele frequencies, mean allelic richness (A), Nei’s gene diversity (H_E_), observed heterozygosity (H_O_), and inbreeding coefficient (F_IS_) using GENETIX 4.05^41^. Normalized allelic richness (Â) and private alleles (PÂ) were also calculated using the approach described in^5^). Values were standardized for a sample size of 20 using 1000 randomizations. Genetic differentiation was estimated between populations using Jost’s D and pairwise F_ST_ (θ^42^) as implemented in the R package “diveRsity”^43^ with 10,000 bootstrap replicates.

Genetic structure was estimated using the ParallelStructure package implementation of STRUCTURE in R^44^ without any prior population assignments. Population clusters for the full microsatellite dataset were tested by setting sequential K from 1 to 10, with 10 replicates for each K, for a total of 2,000,000 Markov Chain Monte Carlo (MCMC) iterations with a 100,000 burn-in each. The optimal number of clusters (K) was selected based on multiple factors. STRUCTURE HARVESTER Web v0.6.94^45^ was used to calculate the ΔK criterion^46^ and a factorial correspondence analysis (FCA) was run as implemented in GENETIX 4.0.5^41^. Initial exploratory analyses showed that the nuclear markers were able to clearly separate the two *Ecklonia* species, with K = 2 being the optimal choice according to the ΔK criterion. With the objective of analyzing genetic structure within each species and the possible hybrids, the dataset was separated into putative species (*E. maxima* and *E. radiata*) and into putative species plus putative hybrids (*E. maxima*, *E. radiata* and putative hybrids from BB and DH). A ParallelStructure analysis was run for each set with the same previous parameters. Runs for the selected optimal Ks were averaged and the results were plotted with CLUMPAK^47^. The number of optimal clusters for *E. maxima* (K = 3) and *E. radiata* (K = 2) was selected based on the ΔK criterion while the best K (K = 4) for the analysis focusing on both species and putative species was selected based on a combination of ΔK and morphological identification of putative hybrids. Results for the three approaches were carefully evaluated manually and are presented here.

The 77 COX1 sequences from South Africa, 127 from Australia and 4 from Oman were used to generate a 207 sequence alignment. Sequence editing, trimming and quality assessment was performed in Geneious 4.8.5 (www.geneious.com), and subsequently aligned with MAFFT v7.470^48^ using the default setting, resulting in a 574 bp alignment. Haplotypes and genetic diversity metrics for each species and region were evaluated as nucleotide diversity (π), number of haplotypes (H), haplotype diversity (Hd), number of private haplotypes (PH), segregating sites (S) and average number of nucleotide differences (K) using DNASP v6^49^. Geographic distribution and genealogy of COX1 haplotypes were mapped and inferred using a median-joining approach as implemented in POPART v1.7^50^. Given the geographic distribution of COX1 haplotypes, genetic pairwise distance between relevant population clusters (see Fig. 1 and Table 2) was calculated in MEGA X^51^.

**Table 2:** Genetic diversity values for the mitochondrial marker COX1 across the different species and regions.

	N	π	H	Hd	PH	S	K
*E. maxima*	45	0.003 ± 0.000	9	0.785 ± 0.035	6	9	1.730
*E. radiata*	159	0.002 ± 0.000	7	0.598 ± 0.001	5	9	1.034
*E. radiata* SA	26	0.004 ± 0.000	6	0.750 ± 0.065	4	11	2.493
*E. radiata* AUS	127	0.001 ± 0.000	3	0.542 ± 0.031	1	1	0.596
*E. spp*	6	0.006 ± 0.002	4	1.00 ± 0.177	4	8	4.000
EMVM	3	0.002 ± 0.000	3	1.00 ± 0.07407	3	2	1.333
ERRB	3	0.000 ± 0.000	1	0.000 ± 0.000	1	0	0
Total	207	0.009 ± 0.000	20	0.889 ± 0.015		24	5.308

*Ecklonia* spp refers to samples from deeper populations, VM and RB. π: nucleotide diversity; H: number of haplotypes; Hd: haplotype diversity, PH: private haplotypes; S: number of segregating sites; K: mean nucleotide differences.

### Species distribution modelling

Species distribution modelling (SDM) was used to estimate niche overlap between the sister species *E. radiata* and *E. maxima*. This approach used Boosted Regression Trees (BRT), a machine learning algorithm with high predictive performance, owing to its ability to fit non-linear relationships and complex interactions between predictor variables, and overfitting reduction through hyperparametrization and forcing of explicit monotonic responses^52,53^.

Biologically meaningful predictors for both species^54,55^ were extracted from the dataset Bio-ORACLE v.2^56^ for the benthic realm (i.e., layers with information for the bottom of the ocean). Predictors were chosen to reflect essential resources (nutrients, as nitrate) and factors affecting physiology (salinity and maximum and minimum temperatures). Presence records for the species were extracted from the fine-tuned dataset of marine forest species^57^. The same number of pseudo-absences as presences were generated for both species in random sites where no presences were recorded^58^. The negative effect of spatial autocorrelation in the models developed for both species was reduced by building a correlogram to estimate the correlation of predictors within increasing distances of occurrence records. Records were then pruned by randomly selecting one record from the group of records found within the minimum distance at which predictors were significantly correlated, e.g.^59^.

A tenfold cross-validation framework using independent blocks^54,60^ was used to find the optimal combination of BRT hyperparameters., specifically, number of trees (from 50 to 1000, step 50 trees), tree complexity (from 1 to 6) and learning rate (0.1, 0.01, 0.005 and 0.001). This also allowed inference of model performances with independent data, as well as their potential for temporal and spatial transferability^54,61^ by using the area under the curve (AUC) of the receiver operating characteristic curve and sensitivity (true positive rate^62^). Models were forced to fit strict monotonic responses, negative for maximum temperature and positive for minimum temperature, nitrate and salinity^53,54,63^. Thermal tolerance limits were determined from individual response functions (i.e., partial dependency functions) produced for maximum temperature, while accounting with the average effect on the models produced by all alternative predictors^54,64^.

### Niche overlap analyses

To assess niche overlap between the two species, the probabilistic method of Swanson et al.^65^ was used. This provides directional estimates of overlap and accounts for the specific distribution of species in niche spaces. The niche region (NR) of each species, inferred with SDM, was defined as the 95% probability region of their multivariate space. The method determined overlap as the probability of an individual from a species being found in the NR of the alternative species, within a Bayesian framework to account for uncertainty.

Niche similarity between species was also addressed following hypothesis testing as proposed by Warren et al.^66^. This approach used two metrics of niche overlap, namely the Warren’s *I*^66^ and the Schoener’s *D* (ranging between 0 and 1, from no overlap to complete overlap), and tested for niche similarity by asking whether niches are more similar to one another than expected by chance. The *D* and *I* values were compared to a null distribution of 104 overlap values produced by linking the niche of one species to a niche generated with random occurrences drawn from the geographic space of the alternative species.

## Results

### Genetic diversity

A total of 181 alleles were identified in 773 individuals at all 8 microsatellite loci. The total number of alleles per locus ranged from 9 to 37, while allele number per population varied between 19 and 71, with 1.54% of missing data. Overall, allelic richness and normalized allelic richness (A, Â) were higher in populations located near the centre of each species distribution, with lower values in populations near the edges (DP, PN and KZ for *E. maxima*, PE, PA and SL for *E. radiata*). The number of private alleles was low overall, with the notable exception of the Australian east coast *E. radiata* populations (CBS, E, PS, SH) and the *E. maxima* populations of Millers Point (MP) and St James (SJ). The deeper population of Rocky Bank (RB) also exhibited low values of allelic richness, while the population of Vema Seamount (VM) had amongst the highest values for *E. maxima*. Expected heterozygosity (H_E_) and observed heterozygosity (H_O_) varied between 0.218–0.687 and 0.108–0.629 respectively and followed a similar pattern to allelic diversity. Most populations showed a significant deficit of observed heterozygosity revealed by significantly high inbreeding coefficient (F_IS_) values (Table 1).

A total of 22 COX1 haplotypes were found (Supplementary Table S3). Haplotype diversity was high within each species and higher for *E. radiata* from South Africa compared to Australia. Nucleotide diversity was low on average due to a few mutations separating each haplotype (Table 2, Fig. 1).

### Genetic structure

Both nuclear and mitochondrial markers clearly separated *Ecklonia maxima* and *E. radiata*, with a few exceptions (Fig. 1, diamond symbols). The first STRUCTURE analysis including the full microsatellite data set revealed two very well supported genetic clusters (Fig. 1C, K = 2) that separated *E. maxima* and *E. radiata*, though some populations had individuals with high admixture levels, most notably VM and ERSDH. Similar results were found in the haplotype network, with *E. maxima* and *E. radiata* clearly separated (Fig. 1B). Both marker types also supported strong geographic patterns of genetic structure for *Ecklonia* in South Africa.

When separating the nuclear data into 4 genetic clusters, subdivision within species becomes more apparent while also highlighting unique characteristics for the deep-water populations and putative hybrids (Fig. 1C, K = 4). Both species are divided into two genetic groups, *E. maxima* having one group that encompasses populations from DP to HK and another from DB to DH and *E. radiata* presenting a genetic split between clusters at DW, where samples show great admixture between clusters. The most remarkable results though are those pertaining to the deeper population from VM and RB, as well as the putative hybrids from BB (EXBB, ErHBOT) and DH (ERSDH). Deeper populations present high admixture between both *E. maxima* clusters and even with *E. radiata* (VM with K4 and RB with K5, Fig. 1C), while putative hybrids from BB show significant admixture between both species and hybrids from DH keep the same pattern as before. The FCA analysis supports the possibility of 4 diversifying clusters, with a separation between *E. radiata* groups, a group with both deeper populations and DH putative hybrids at the edges of the major *E. maxima* cluster and a diverging group made up of the putative BB hydrids (Fig. 1D).

A second level of subpopulations was revealed by the analyses focusing on each specific species. *Ecklonia maxima* was further divided into 3 genetic clusters, with clear geographic distributions for each one (Fig. 1A,C). The K1 cluster ranged from Diaz Point in Namibia (DP) southwards to Hondeklipbaai (HK), K2 from Doringbaai (DB) to Cape Agulhas (CA), with K3 found only in De Hoop (DH). Haplotype distributions for *E. maxima* support similar geographic patterns (Fig. 1A). A western group comprised of a private haplotype at Diaz Point (H12), closely related to the haplotype (H11) was found in locations sharing the same nuclear cluster (PN up to HK) and in populations further south (DB, KM). Another private haplotype (H13) was found in this region, only in Jacobs Bay (JB). A second geographic group was found between populations from Millers Point (MP) to Quoin Point (QP), with haplotype H10 being shared and corresponding to nuclear cluster K2. Finally, a third group of closely related haplogroups (H19, H20) is distributed from Muizenberg (MZ) to De Hoop (DH), with a private haplogroup present in Cape Agulhas (H21).

Mitochondrial DNA from deeper populations diverged from coastal ones in both the haplotype network (Fig. 1B) and in terms of average genetic distance (Table 3). Vema Seamount (VM) and Rocky Bank (RB) presented private haplotypes that diverged from both *E. maxima* and *E. radiata* (H15–17 and H22, respectively). VM haplogroups were closely related to those found in the Cape Town region, (i.e. H10 at Millers Point, Bordjiesrif and Saint James; H14 at Muizenberg). In contrast, putative hybrids from deeper waters at BB (ERHBOT, EXBB) were found with *E. radiata* haplotype (H5), showing incongruence between nuclear and mitochondrial markers.

**Table 3:**
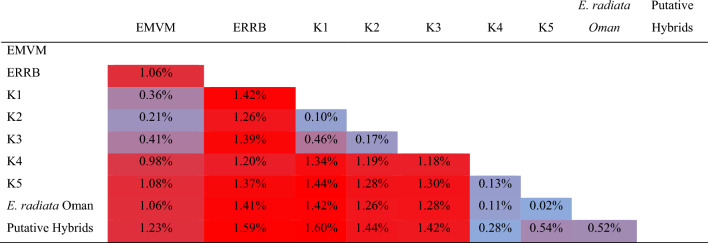
Pairwise mitochondrial sequence divergence (%) at the COX1 locus, between species and geographic groups of *Ecklonia* based on COX1 sequences.

Populations were grouped based on nuclear genetic clusters (*E. maxima* K1, K2, K3 and *E. radiata* K4 and K5) and populations of interest (Putative Hybrids: EXBB, ERHBOT, and deeper populations of VM, RB and extinct Omani populations).

In the same STRUCTURE analysis, *E. radiata* was split into two genetic clusters with a clear geographic break (Fig. 1C). A southern K4 cluster grouped populations from Port Elizabeth (PE) to Dwesa (DW) and a second K5 cluster grouped populations from northeastern South Africa (MK and SL), Mozambique (MOZ) and Australia (AUS). The distribution of haplotypes in *E. radiata* were not completely consistent with these discrete nuclear genetic clusters. One haplotype (H1) is shared across most of the distribution range of *E. radiata*, spanning from Australia (AUS) to Port Elizabeth (PE). Only four populations do not share this haplotype: St Lucia (SL), Three Sisters (TS), Port Alfred (PA) and Abrolhos Island (AB) displayed private haplotypes. Additionally, a unique haplotype was found in Oman. A single haplotype (H5) is shared between Port Elizabeth and De Hoop. The sample from De Hoop (ERSDH03) with this haplotype exhibited a high level of genome admixture from *E. maxima* and *E. radiata* in the K = 2 STRUCTURE analysis and was placed within the *E. radiata* cluster in the FCA analysis (Fig. 1A–D). Geographic patterns for both species are corroborated by population differentiation as measured with F_ST_ and Jost’s D (Supplementary Table S4).

### Specimen identification

The combination of nuclear and mitochondrial markers was essential for the identification of individuals sampled in interspecific contact zones and in deeper offshore populations because there were clear instances of mito-nuclear discordance as well as between morphological and genetic species identification. Deeper individuals in BB (ERHBOT, EXBB) had *E. radiata* haplotypes (H5) but clustered within *E. maxima* in the STRUCTURE analysis with K = 2, showed great admixture between species at K = 4 and were a diverging group in the FCA (Fig. 1B–D). A similar nuclear-mitochondrial discordance was found for one individual from the De Hoop contact zone (ERSDH03). De Hoop clustered as a unique genetic group within *E. maxima* (K3, Fig. 1C) but one individual of this nuclear cluster had an *E. radiata* haplotype (H5, Fig. 1B). Genetic distances between these individuals with discordant genomes and other *Ecklonia* populations confirm them having a nuclear genome of *E. maxima* and mitochondrial genome of *E. radiata*, with low F_ST_ and Jost’s D relative to *E. maxima* and inversely low pairwise genetic distance to *E. radiata* for COX1. Both observations suggest hybridization in the two contact zones. Most samples from De Hoop were conclusively recovered as *E. maxima* with both nuclear and mitochondrial markers, regardless of their morphology (Fig. 1). The only exceptions were the 6 individuals labeled as ERSDH, for which nuclear markers showed high admixture (Fig. 1C), low genetic distances from both species (Supplementary Table S4), and this population had the only sequenced individual with an *E. radiata* haplotype (Fig. 1A,B; H5). Similar mito-nuclear discordance was found for the atypical samples from Bordjiesrif (EXBB, ERHBOT), which were recovered as part of the major *E. maxima* nuclear cluster (Fig. 1C, K  = 2) but possessed a unique *E. radiata* haplotype (Fig. 1A,B; H5).

Deeper populations from Vema Seamount (VM) and Rocky Bank (RB) produced distinct results. While both were recovered as *E. maxima* in the K = 2 analysis, with both having high levels of admixture between *E. maxima* clusters and some with *E. radiata* in the K = 4 analysis. The mtDNA analyses revealed unique haplotypes for VM which are closely related to *E. maxima* (H15–17, Fig. 1B). In contrast, the haplotype from RB was intermediate with respect to the two species, and pairwise genetic distances, both between species and geographic groups, confirm this. The *Ecklonia* sp. from RB is divergent from both *E. maxima* and *E. radiata* (Table 3), possibly representing a distinct *Ecklonia* lineage.

### Species distribution models

**Figure 2:**
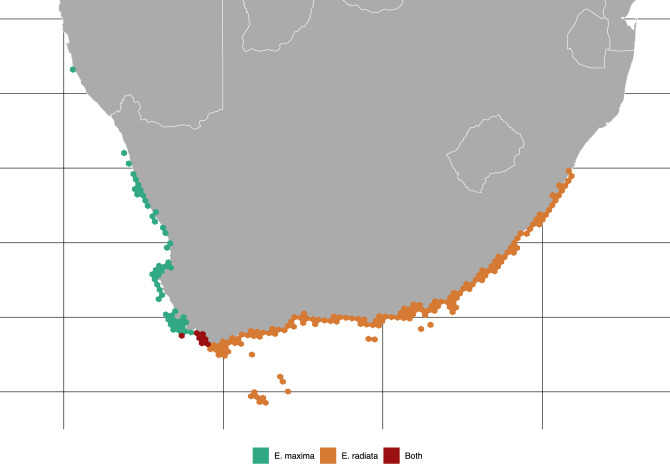
Potential distribution of *Ecklonia radiata* and *Ecklonia maxima* predicted with Species Distribution Modelling. Predictions aggregated in equal area hexagons for better visualization. The model correctly predicted the presence of both species in the Buffels Bay area but was not able to recover the same result for De Hoop.

The marine forests dataset retrieved a total of 37,105 and 466 occurrence records for *E. radiata* and *E. maxima* (Table S5), which after pruning resulted in 272 records. The models using the best combination of hyperparameters showed good potential for transferability (Cross-validation AUC > 0.83; Cross-validation Sensitivities > 0.92; Table S5) and the final predictions largely matched the known distribution of the species (Final AUC > 0.9; Final Sensitivities > 0.97; Table S5; Fig. 2), with the exception of *E. maxima* presence at De Hoop.

### Niche overlap analyses

The probability of an individual of *E. radiata* being found within the ecological niche of *E. maxima* was 29.72%, while the contrary, finding an individual of *E. maxima* within the niche of *E. radiata* was 66.63% (Fig. S1). Species distribution modelling showed *E. radiata* to have a higher tolerance to maximum temperature when compared to *E. maxima* (21.3 °C vs. 26.2 °C; Fig. S2).

The similarity test did not reject the null hypothesis of the background test; thus, indicating that both niches are less similar than expected from random niches drawn (Tables S6 and S7). Overall, the Bayesian probabilistic niche overlap analyses and the similarity tests provide evidence for niche differentiation between *E. radiata* and *E. maxima,* while indicating that *E. radiata* has a much broader ecological niche than *E. maxima.* Putative hybrids EXBB and ER-h-BOT niche was more likely to overlap with that of *E. radiata*, the same being true for the deep samplings from VM and RB, while ERSDH niche was more similar to that of *E. maxima* (Table S8).

## Discussion

Our results reveal the presence of discrete genetic groups within both *Ecklonia* species along distinct geographical sectors and depths of the southern African coastlines spanning from Namibia to Mozambique. Besides general population differentiation, the molecular species assignment in some cases differs from expectations based on morphology. Furthermore, a few instances of mito-nuclear incongruence were found in the contact zones between the two *Ecklonia* species, accompanied by the presence of atypical morphological traits, suggesting the occurrence of species hybridization. Notably our analysis of nuclear and mitochondrial data indicates the existence of a putative third *Ecklonia* lineage at Rocky Bank, separate from coastal populations. Below we discuss the implications of these findings for the characterization of southern African *Ecklonia*.

### Phylogeography of southern African *Ecklonia*

*Ecklonia maxima* and *E. radiata* were confirmed to have distinct and mostly discrete distributions along the coast of southern Africa. The northernmost extant distribution of *E. maxima* was confirmed at Diaz Point (Lüderitz) in Namibia, extending along the west coast of South Africa as far east as Cape Agulhas. We also discovered morphologically atypical *E. maxima* populations in deep offshore habitats (Vema Seamount) for the first time. Additionally, our molecular data confirms previous reports of *E. maxima* in Koppie Alleen, in the De Hoop Nature Reserve^8,21^ confirming this site as a contact zone between the species.

*Ecklonia maxima* showed marked genetic structure along its distribution, with three nuclear genetic groups and 12 mitochondrial haplotypes. The geographic distribution of this diversity is very pronounced, in line with some previously described phylogeographic breaks on the coast of South Africa^67–70^. Cluster K1 (with haplotypes H11 and H12) was found in the cool-temperate region of Namaqua, from Namibia southwards to the southwestern Cape bioregion^71,72^. The break between nuclear clusters K1 and K2 between Hondeklipbaai (HB) and Doringbaai (DB) is similar to that described for another dominant kelp species, *Laminaria pallida*^73^. The private haplotype of *E. maxima* in southern Namibia, and larger population differentiation values between this and the closest South African populations suggest limited connectivity, although the possibility that sampling was insufficient to detect most haplotypes is an alternative explanation. The formation of cyclonic eddies due to the convergence of currents near Lüderitz^74,75^ may contribute to the isolation of this population. The oceanographic conditions and marginal location of this population is also a possible cause for localized, non-random mating, thereby explaining the high inbreeding coefficient.

Cluster K2 is found in the southwestern Cape bioregion^71,72^, along with haplotypes H10–11, H13–14, and H18–20, respectively. This is where most of the diversity within *E. maxima* is concentrated, with populations displaying the highest overall levels of allelic richness, gene diversity and observed heterozygosity (Table 1). A small number of private alleles, low F_ST_ values and haplotype sharing (H10) point to significant population connectivity in the region. The Benguela current, coupled with rare long distance dispersal events involving fertile floating thalli^76^, can also explain the presence of K2 and H11 from Kommetjie (KM) up to Doringbaai (DB).

The dominant current in this region also helps to explain the presence of *E. maxima* in Vema Seamount and its genetic nature. Agulhas rings eddies shed by the Agulhas Current move towards the South Atlantic, and are known to reach Vema Seamount^77^. Together with the Benguela Current, favoring northern and offshore dispersal, it is very likely that gametophytes or rafting individuals of both *E. maxima* genetic clusters reached Vema Seamount and settled there. Indeed, *E. maxima* drift plants have been found up to 600 km east of its distribution^8,32^. However, the high nuclear divergence of this group and the presence of 3 private haplotypes in the area (despite the possibility that sampling was insufficient to detect most haplotypes) is striking and suggests an ancient and rare colonization of this region, where it has evolved independently for a substantial period of isolation from coastal populations.

Cape Agulhas acts as an important phylogeographic break for *E. maxima*, marking the split between nuclear clusters K2 and K3 and harboring a private haplotype. This break is well-established and marks the transition between the Southwestern Cape and the warm-temperate Agulhas bioregion^67,69,70,78^. The existence of a unique nuclear cluster (K3) at De Hoop suggests the isolation of this population. Currents near Cape Agulhas are complex, with strong westwards flow and weak and variable flow to the east^79–81^, connectivity between De Hoop and the nearest sampled populations (CA, PE) can be relatively low. Adding to this, De Hoop is mostly dominated by warm-water algae^82^ and species distribution modelling only predicted the presence of the warm-adapted *E. radiata*, meaning local conditions could be driving the divergence of this cold-adapted *E. maxima* cluster.

Genetic structure in *E. radiata* was less pronounced compared to *E. maxima*. Nonetheless, the two genetic clusters recovered have well-defined geographical distributions. Cluster K4 is distributed along the eastern limit of the warm-temperate Agulhas region and in the Wild Coast transition zone^83–85^, extending up to Mkhambathi, near the border of Eastern Cape and KwaZulu Natal provinces. While the boundary between the warm-temperate Agulhas region and the Natal bioregion has been difficult to define^67,68,70,83^, the break between nuclear clusters K4 and K5 corresponds to the northern limit of the Wild Coast transition region^67,83,84^. Cluster K5 has a sub-tropical to tropical distribution^86–88^, extending to Mozambique, and even to the eastern coast of Australia. The shared nuclear genetic cluster between South Africa and Australia is congruent with common *E. radiata* COX1 haplotypes between these regions^12,89^. Indeed, broad scale haplotype sharing (H1, from South Africa to Oman, western and eastern Australia, and Tasmania), suggests recent and/or rapid expansion in *E. radiata*. The presence of *E. radiata* in tropical and sub-tropical locations such as Mozambique, St Lucia and Moreton Island, Australia is noteworthy and may be facilitated by pockets of cooler deep reef oceanic habitats where light and temperature conditions are suitable for the species^12,29^, as known for other kelps^5,6,76,90,91^. These deeper populations might have played a critical role in the dispersal and equatorial crossing of *E. radiata*, as evidenced by extinct populations in Oman^12^. Genetic clustering and the haplotype distribution of *E. radiata* in South Africa can be attributed to the effect of the Agulhas Current, promoting gene flow along the eastern shore into suitable habitats, and subsequent genetic divergence through drift in small, isolated populations. Genetic pairwise differences between populations are mostly consistent with the previously described patterns, with F_ST_ and Jost’s D values increasing with population distance from southwest to northeast, and a significant increase at Mkhambathi, marking a phylogeographic break for *E. radiata*.

### Introgression and speciation along the southern African coast

Our results challenge some previous concepts about southern African *Ecklonia* and further explain some unresolved questions. Molecular data confirmed reports of *E. maxima* at Koppie Alleen, in the De Hoop Nature Reserve^8,21^ and suggests it is currently the dominant kelp at this location. Although in general, both nuclear and mitochondrial markers identified only *E. maxima* in this region, a subset of individuals had clearly admixed nuclear genomes and one individual had an *E. radiata* haplotype. A similar pattern of mito-nuclear discordance was found for the atypical deeper samples from Bordjiesrif in the Cape Peninsula, which were part of the major *E. maxima* nuclear cluster but on further analysis presented a highly admixtured genotype and a unique *E. radiata* haplotype. These cases of marker discordance suggest that hybridization occurs when the species are in contact, as shown in nature between other *Ecklonia* species^23^ and experimentally between *E. maxima* and *E. radiata*^16^.

Multiple hybridization events have likely occurred, because putative hybrids from De Hoop have high admixture and do not diverge much from either parental species, while the atypical samples from Bordjiesrif seem to be evolving in isolation. The latter possess a unique haplotype (H5) and forms a unique nuclear cluster in the FCA. Additionaly, pairwise genetic distances were relatively high between each of these populations with each other and with all others, further indicating their isolation and the lack of gene flow with other *Ecklonia* genetic entities. Although we cannot exclude that geographical isolation and/or depth at Bordjiesrif might be driving this isolation, differences between putative hybrid populations here and at De Hoop may have a temporal basis, with hybridization being more recent in the range contact zone of De Hoop than in the deeper distinct population of Bordjiesrif. Alternatively, a process of genetic hitchhiking might also be responsible for the increased divergence of these populations, facilitating the fixation of the results from an ancient hybridization.

The frequency and extent of hybridization between these species is an important question. Hybridization may result in heterosis (increased fitness compared to one or both parental species^92^, as recently demonstrated in some kelp species^18,93^. Experimental *E. maxima/E. radiata* hybrids showed broad thermal optima for growth^16^, possibly giving them an adaptive advantage. Our niche similarity modelling indicated that putative hybrids from Bordjiesrif are more likely to occupy a niche more similar to *E. radiata* than *E. maxima*, while putative hybrids from De Hoop have a niche overlapping that of *E. maxima*. Scenarios in which more adapted hybrids outcompete the parental species may lead to the loss of unique genetic diversity.

The presence of a distinct genetic group in the De Hoop region indicates that these populations have had time to diverge from the nearest sampled *E. maxima* and thus may have been established for longer than previously suspected^8^. *Ecklonia* species exhibit considerable morphological plasticity, making species identification and delimitation based on morphological traits challenging^23,25,94^. However, while morphological plasticity has been widely documented in *E. radiata*, leading to multiple taxonomic updates and the synonymization of other species with *E. radiata*^29,94–97^, *E. maxima* has been considered morphologically stable across most of its distribution^27^. Recent modeling studies have demonstrated size reduction in *E. maxima* from shallow populations exposed to higher temperatures^98^. Changes in stipe morphology, such as hollow versus solid, correlating with abiotic factors were described in another South African kelp, *Laminaria pallida*^27,98^, previously considered a diagnostic character separating *L. pallida* and (now synonymized) *Laminaria schinzii*^32^*.* Lower growth rates have been associated with nutrient-poor conditions^99–101^, which could be expected in the warm shallows and intertidal pools of De Hoop. Furthermore, although species distribution modelling provided evidence for niche differentiation between the species, the presence of *E. maxima* at De Hoop was not predicted. Changes in morphology due to abiotic factors and/or hybridization may therefore have played a role in the appearance of atypical *E. maxima* individuals at De Hoop.

Finally, we provide evidence for the existence of a likely third lineage of *Ecklonia* along the South African coast. Although individuals from the deep Rocky Bank population were part of *E. maxima* by K = 2 (species) clustering and were at the edge of the *E. maxima* cluster in FCA analysis, samples from Rocky Bank had a unique mixed genotype when considering a K = 4. The mitochondrial haplotype from Rocky Bank (H22) was intermediate and divergent from both species, while pairwise mitochondrial genetic distances between Rocky Bank *Ecklonia* and either *E. maxima* or *E. radiata* were comparable to intraspecific divergence in *Ecklonia*^21,25,35^. Further support for this distinct genetic entity comes from elevated pairwise nuclear genetic distances between Rocky Bank and populations of either species. However, it remains unclear whether the RB population represents a previously described species or a new diverging species. Further investigation using additional molecular markers and multispecies coalescent approaches could provide a better insight into the nature of the divergence between the South African *Ecklonia* lineages and their delimitations.

An important discovery is the significant genetic diversity and divergence from coastal *Ecklonia* observed in populations sampled from deeper habitats at Vema Seamount (VM) and Rocky Bank (RB), rendering them a critical genetic component of the genus in Southern Africa. These and previously identified deep populations^12,29,102^ add to the growing realization of deep reefs as important genetic refugia^5,6,76,91,103,104^. Understanding the functional genetic divergence between coastal and deeper populations is of utmost importance considering the predicted trends and pressures on this unique biodiversity^105^.

## Conclusions

We present the first comprehensive analysis of genetic diversity and population structure of the kelps *Ecklonia maxima* and *E. radiata* in southern Africa. Our findings confirm the presence of a deep *E. maxima* population located 1000 km offshore of the west coast of South Africa, on Vema Seamount. Furthermore, our results suggest an earlier-than-presumed expansion of *E. maxima* eastward to the De Hoop, potentially hindered in detection by morphological plasticity and hybridization in contact zones. This shift may have led to the replacement of the previously dominant *E. radiata* by *E. maxima* in the region. The known and predicted distribution of *E. radiata* was confirmed, notably deep populations in the Natal and Delagoa bioregions in South Africa and Mozambique. Intriguingly, our data also reveal the existence of a possible third *Ecklonia* lineage in the deep population of Rocky Bank, where both nuclear and mitochondrial markers exhibit unexpectedly high levels of divergence. These results underscore the importance of molecular data in identifying cryptic diversity, particularly in morphologically plastic genera capable of hybridization^23,25,106,107^*.* Further analysis that integrates taxonomy and genetics will be key for understanding the complex dynamics of hybridization in a region where ocean conditions are predicted to undergo complex and unique changes.

### Supplementary Information

Supplementary Information.

## Data Availability

Genotypic data were deposited in a Figshare repository @ https://figshare.com/s/6a207ffc3d716b117c6d. New COXI sequences were deposited in GenBank under the accession numbers OR41340 to OR413508, OR413573 and PP209592 to PP209593 (available on Table S2 of the Supplementary Information).
