# Supplementary material for: Cryptic diversity in southern African kelp
Source: Sci Rep. 2024 May 14;14:11071. doi: 10.1038/s41598-024-61336-4 (PMC11093989; doi:10.1038/s41598-024-61336-4)
Supplement: Supplementary file 1 — Supplementary Information. [file 41598_2024_61336_MOESM1_ESM.pdf]

Cryptic diversity in southern African kelp

Pedro Madeira†, Maggie M. Reddy†, Jorge Assis, John J. Bolton, Mark D. Rothman, Robert J. Anderson, Lineekela Kandjengo, Anja Kreiner, Melinda A. Coleman, Thomas Wernberg, Olivier De Clerck, Frederik Leliaert, Salomão Bandeira, Abdul M. Ada, João Neiva, Gareth A. Pearson, Ester A. Serrão

† These authors equally contributed to this work

Supplementary Information

Material and Methods

Sampling, DNA extraction, genotyping and sequencing

Table S1 – Primer sequences (A), PCR reagents (B) and conditions (C) for the amplification of microsatellite and COX1 primers

A)Microsatellite primers

Fluoroph	Name	Sequence	Source
ATTO550	EC03-F	CCCATCAACAGGACAACGAAG	Itou et al. 2012
	EC03-R	GTTTCTTGCCGGCGTGAGTAGGAGTTC	Itou et al. 2012
ATTO565	EC07-F	CGTTCCAGACATCAGACACA	Itou et al. 2012
	EC07-R	GTTTCTTTGGTAGTCAATGGCATAGG	Itou et al. 2012
HEX	EC10-F	GTCCCCACATCCGAAAGGTAAC	Itou et al. 2012
	EC10-R	GTTTCTTCAAGCGGTAAGGGTGTCAAG	Itou et al. 2012
FAM	EC12-F	TCCTGGAACTCCGCGATCTTC	Itou et al. 2012
	EC12-R	GTTTCTTGCAATTATAGAGGGCGGTGAA	Itou et al. 2012
FAM	Eradic01-F	ATTCGAATTGTGCGAGATCC	Akita et al. 2018
	Eradic01-R	CTTGGTCGGCAAAAGTCATT	Akita et al. 2018
ATTO565	Eradic04-F	ACGTCTTCTGGTGTGCTGTG	Akita et al. 2018
	Eradic04-R	AAATGTCGTCAAATGTGGCA	Akita et al. 2018
HEX	Eradic05-F	GAAGATGCCCCTTGAGAACA	Akita et al. 2018
	Eradic05-R	AAGGAGTCCCACTGAGCAGA	Akita et al. 2018
ATTO550	Eradic07-F	TATGTCACGTCTCTGGCTCG	Akita et al. 2018
	Eradic07-R	TCGGACAAGTCTCCTCAACC	Akita et al. 2018
ATTO565	Eradic09-F	GGTAAGGCGATGGACGATAA	Akita et al. 2018
	Eradic09-R	TCTTACGGTAGCCCGAGAAA	Akita et al. 2018

COX1 primers

Primer	5'→3'	Source
GazF2	CCAACCAYAAAGATATWGGTAC	Lane et al. 2007
GazR2	GGATGACCAAARAACCAAAA	Lane et al. 2007

B)PCR mix

EC03 and EC10 (Itou et al., 2012)

**Mix_2mM Mg**	**[ ] final**	**1x (µL)**
H_2O	-	4,35
Buffer (5x)	1x	3
$MgCl_2$ (25mM)	2mM	1,2
dNTP's (2mM each)	0.07mM each	0,5
Primer F (10µM)	0.25µM	0,375
Primer R (10µM)	0.25µM	0,375
GoTaq G2 (5U/µL)	1U	0,2
DNA (1:100) Nucleospin	-	5

EC07 and EC12 (Itou e al. 2012)

**Mix_2mM Mg**	**[ ] final**	**1x (µL)**
H_2O	-	3,6
Buffer (5x)	1x	3
$MgCl_2$ (25mM)	2mM	1,2
dNTP's (2mM each)	0.07mM each	0,5
Primer F (10µM)	0.5µM	0,75
Primer R (10µM)	0.5µM	0,75
GoTaq G2 (5U/µL)	1U	0,2
DNA (1:100) Nucleospin	-	5

Erad01, Erad 04, Erad 05 and Erad09 (Akita et al., 2018)

**Mix_2mM Mg**	**[ ] final**	**1x (µL)**
H_2O	-	3,6
Buffer (5x)	1x	3
$MgCl_2$ (25mM)	2mM	1,2
dNTP's (2mM each)	0.07mM each	0,5
Primer F (10µM)	0.5µM	0,75
Primer R (10µM)	0.5µM	0,75
GoTaq G2 (5U/µL)	1U	0,2
DNA (1:100) Nucleospin	-	5

COX1

Mix 2mM Mg	[ ] final	1x (µL)
H_2O	-	5,95
Buffer (5x)	1x	4
$MgCl_2$ (25mM)	2mM	1,6
dNTP's (2mM each)	0.125mM each	1,25
GazF2 (10µM)	0.5µM	1
GazR2 (10µM)	0.5µM	1
GoTaq G2 (5U/µL)	1U	0,2
DNA (1:100) Nucleospin	-	5

C)PCR conditions

EC03, EC07, EC10 and EC12

Steps	T (ºC)	Time	Cycles
**Denaturation**	95	5'	1
**Denaturation**	95	30''	35
Annealing	57	30''	
**Extension**	72	30''	
**Extension**	72	10'	1

Erad01, Erad04, Erad05, Erad09

Steps	T (ºC)	Time	Cycles
**Denaturation**	95	5'	1
**Denaturation**	95	30''	35
Annealing	58	30''	
**Extension**	72	30''	
**Extension**	72	10'	1

COX1

Steps	T (ºC)	Time	Cycles
**Denaturation**	95	5’	
**Denaturation**	95	30’’	35
Annealing	50	45’’	
**Extension**	72	1’’	
**Extension**	72	10’	

Table S2 – Details of sequenced individuals and sequences obtained from GenBank.

Species	Code	N	Location	GB Accession	Source
Ecklonia maxima	EMDP01-03	3	Diaz Point, Namibia	OR421573; OR413450-1	This paper
Ecklonia maxima	EMKM01;05;10	3	Kommetjie, South Africa	OR413452-4	This paper
Ecklonia maxima	EMHK01;07;10	3	Hondeklipbaai, South Africa	OR413455-7	This paper
Ecklonia maxima	EMJB01;03;18	3	Jacob's Bay, South Africa	OR412458-60	This paper
Ecklonia maxima	EMKZ01;02;07	3	Kleinzee, South Africa	OR412461-3	This paper
Ecklonia maxima	EMDB01;02;05	3	Doringbaai, South Africa	OR413480-2	This paper
Ecklonia maxima	EMPN01;02;07	3	Port Nolloth, South Africa	OR413469-71	This paper
Ecklonia maxima	EMMP01;02	2	Millers Point, South Africa	OR413464-5	This paper
Ecklonia maxima	EMBB01;02;11	3	Buffels Bay, South Africa	OR413480-2	This paper
Ecklonia maxima	EMBOT01-03	3	Buffels Bay, South Africa	OR413483-5	This paper
Ecklonia maxima	EMSJ01;03	2	St. James False Bay, South Africa	OR413475-6	This paper
Ecklonia maxima	EMMUI01;03;24	3	Muizenberg, South Africa	OR413466-8	This paper
Ecklonia maxima	EMCA01-03	3	Cape Agulhas, South Africa	OR413486-8	This paper
Ecklonia maxima	EMQP01-03	3	Quoin Point, South Africa	OR413472-4	This paper
Ecklonia maxima	ERDH01;03;10	3	De Hoop, South Africa	OR413494-6	This paper
Ecklonia maxima	EMDH01;04	2	De Hoop, South Africa	OR413492-3	This paper
Ecklonia maxima	ERHOO01-03	3	De Hoop, South Africa	OR413497-9	This paper
Ecklonia sp.	EMVM02;03;04	3	Vema Seamount, South Africa	OR413477-9	This paper
Ecklonia sp.	ERRB03;05;07	3	Rocky Bank, South Africa	OR413505;PP209592-3	This paper
Ecklonia radiata	ERPA01;03;10	3	Port Alfred, South Africa	OR413500-2	This paper
Ecklonia radiata	ERTS01;05	2	Three Sisters, South Africa	OR413506-7	This paper
Ecklonia radiata	ERPE02;05	2	Port Elizabeth, South Africa	OR413503-4	This paper
Ecklonia radiata	ESTL02	1	Santa Lucia, South Africa	OR413508	This paper
Ecklonia radiata	ERSDH03	1	De Hoop, South Africa	OM650133	This paper
Ecklonia radiata	EhBOT01-03	3	Buffels Bay South Africa	OM650140-2	This paper
Ecklonia radiata	EXBB02;05;13	3	Buffels Bay, South Africa	OM650135-7	This paper
Ecklonia radiata	ErHLU01-02	2	Hluleka, South Africa	OM650126-7	This paper
Ecklonia radiata	ERDW01,02,04	3	Dwesa, South Africa	OM650128-30	This paper
Ecklonia radiata	ERMK01-03	3	Mkhambathi, South Africa	OM650123-5	This paper
Ecklonia radiata	EMOZT03; EMOZDRS03; EMOZDR03	3	Zavora, Mozambique	OM650143-5	This paper
Ecklonia radiata	AUS_DD01-05	5	Drumsticks	KT15799-803	(Durrant et al., 2015)
Ecklonia radiata	AUS_CR01-04	4	CallalaReef	KT158795-8	(Durrant et al., 2015)
Ecklonia radiata	AUS_OT01-04	4	OuterTubes	KT158851-4	(Durrant et al., 2015)
Ecklonia radiata	AUS_EI01-04	4	ErithIsland	KT158808-11	(Durrant et al., 2015)
Ecklonia radiata	AUS_SC01-05	5	SquallyCove	KT158857-61	(Durrant et al., 2015)

Species	Code	N	Location	GB Accession	Source
Ecklonia radiata	AUS_O01-03	3	Oakhampton	KT158848-50	(Durrant et al., 2015)
Ecklonia radiata	AUS_PH01-02	5	PointHolme	KT158855-6	(Durrant et al., 2015)
Ecklonia radiata	AUS_DP01-04	4	DennesPoint01-04	KT158804-7	(Durrant et al., 2015)
Ecklonia radiata	AUS_BB01-05	5	BlackmanBay01-05	KT158785-9	(Durrant et al., 2015)
Ecklonia radiata	AUS_GB01-03	3	GreenBluff01-03	KT158822-4	(Durrant et al., 2015)
Ecklonia radiata	AUS_HI01-04	4	HuonIsland01-04	KT158828-31	(Durrant et al., 2015)
Ecklonia radiata	AUS_LP01-04	4	LucasPoint01-04	KT158836-39	(Durrant et al., 2015)
Ecklonia radiata	AUS_FP01-05	5	FarrellPoint01-05	KT158817-19	(Durrant et al., 2015)
Ecklonia radiata	AUS_IS01-04	3	InnerSaddle01-04	KT158832-34	(Durrant et al., 2015)
Ecklonia radiata	AUS_MBI01-03	3	MuttonbirdIsland01-03	KT158842.1	(Durrant et al., 2015)
Ecklonia radiata	AUS_SN01-03	3	SnapperNorth01-03	KT158862-4	(Durrant et al. 2015)
Ecklonia radiata	AUS_FI01-05	5	FlatIrons01-05	KT158812-6	(Durrant et al., 2015)
Ecklonia radiata	AUS_GI01-03	3	GoatIsland01-03	KT158825-7	(Durrant et al., 2015)
Ecklonia radiata	AUS_BR01-05	5	BaudinRocks01-05	KT158790-4	(Durrant et al., 2015)
Ecklonia radiata	AUS_MS01-05	5	MypongaSouth01-05	KT158843-7	(Durrant et al., 2015)
Ecklonia radiata	AUS_A16-27	11	Albany16-26	OL456958; OL456963-71	(Coleman et al., 2022)
Ecklonia radiata	AUS_G01;37;39;40;45;49;51;52;53;56;60	11	Geraldton01;37;39;40;45;49;51;52;53;56;60	OL456972-81	(Coleman et al., 2022)
Ecklonia radiata	AUS_JB10;11;15;17;18;19;21;23;26;27;54	11	JurienBay10;11;15;17;18;19;21;23;26;27;54	OL456962- OL456982-91	(Coleman et al., 2022)
Ecklonia radiata	AB1-10_AUS	10	AbrolhosIsland01-10	OL456948-57	(Coleman et al., 2022)
Ecklonia radiata	OM_BPT; OM_MICH613469; OM_MICH613506-7_	4	Oman	OL456959; OL456992-4	(Coleman et al., 2022)

Table S3 – Haplotype data for COX1

H1 n=88	AUS_A27 AUS_A16 AUS_A17 AUS_A18 AUS_A19 AUS_A20 AUS_A21 AUS_A22 AUS_A23 AUS_A24 AUS_A26 AUS_BR01 AUS_BR02 AUS_BR03 AUS_BR04 AUS_BR05 AUS_CR01 AUS_CR02 AUS_CR03 AUS_CR04 AUS_DD01 AUS_DD02 AUS_DD03 AUS_DD04 AUS_DD05 AUS_FI01 AUS_FI02 AUS_FI03 AUS_FI04 AUS_FI05 AUS_FP02 AUS_FP03 AUS_FP04 AUS_FP05 AUS_FP01 AUS_G1 AUS_G37 AUS_G39 AUS_G40 AUS_G45 AUS_G49 AUS_G51 AUS_G52 AUS_G53 AUS_G56 AUS_G60 AUS_GI01 AUS_GI02 AUS_GI03 AUS_IS04 AUS_JB10 AUS_JB11 AUS_JB15 AUS_JB17 AUS_JB18 AUS_JB19 AUS_JB21 AUS_JB23 AUS_JB26 AUS_JB27 AUS_JB54 AUS_MS01 AUS_MS02 AUS_MS03 AUS_MS04 AUS_MS05 AUS_OT01 AUS_OT02 AUS_OT03 AUS_OT04 AUS_SN01 AUS_SN03 AUS_SN03 EMOZDR EMOZDRS EMOZT ERDW01 ERDW02 ERDW04 ErHLU01 ErHLU02 ERMK01 ERMK02 ERMK03 ERPE02 OM_MICH613469 OM_MICH613506 OM_MICH613507
H2 n=10	AB10_AUS AB1_AUS AB2_AUS AB3_AUS AB4_AUS AB5_AUS AB6_AUS AB7_AUS AB8_AUS AB9_AUS
H3 n=40	AUS_BB01 AUS_BB02 AUS_BB03 AUS_BB04 AUS_BB05 AUS_DP01 AUS_DP02 AUS_DP03 AUS_DP04 AUS_EI01 AUS_EI02 AUS_EI03 AUS_EI04 AUS_GB01 AUS_GB02 AUS_GB03 AUS_HI01 AUS_HI02 AUS_HI03 AUS_HI04 AUS_IS01 AUS_IS02 AUS_IS03 AUS_LP01 AUS_LP02 AUS_LP03 AUS_LP04 AUS_MBI01 AUS_MBI02 AUS_MBI03 AUS_O01 AUS_O02 AUS_O03 AUS_PH01 AUS_PH02 AUS_SC01 AUS_SC03 AUS_SC04 AUS_SC04 AUS_SC05
H4 n=1	OM_BPT
H5 n=6	EhBOT01 EhBOT02 EhBOT03 EXBB02 EXBB05 EXBB13
H6 n=2	ERPE05 ERSDH03
H7 n=2	ERTS01 ERTS05
H8 n=3	ERPA01 ERPA03 ERPA10
H9 n=1	ESTL02
H10 n=11	EMBB01 EMBB03 EMBOT01 EMBOT02 EMBOT03 EMMP01 EMMP02 EMQP01 EMQP02 EMSJ01 EMSJ03
H11 n=17	EMDB01 EMDB02 EMDB05 EMHK01 EMHK07 EMHK10 EMJB01 EMJB18 EMKM01 EMKM05 EMKM10 EMKZ01 EMKZ02 EMKZ07 EMPN01 EMPN02 EMPN07
H12 n=3	EMDP01 EMDP02 EMDP03
H13 n=1	EMJB03
H14 n=1	EMMUI01
H15 n=1	EMVM02
H16 n=1	EMVM04
H17 n=1	EMVM03
H18 n=1	EMBB11
H19 n=10	EMDH01 EMDH04 EMMUI24 EMQP03 ERDH01 ERDH03 ERDH10 ERHOO01 ERHOO02 ERHOO03
H20 n=1	EMMUI03
H21 n=3	EMCA01 EMCA02 EMCA03
H22 n=3	ERRB03 ERRB05 ERRB07

Table S4 – Genetic distance between South African Ecklonia populations as measured by F_{st} (A) and Jost'D (B)

A)

Samples	EMDP	EMPN	EMKZ	EMHK	EMDB	EMJB	EMKM	EMMP	EMVM	EMSJ	Em-MUI	EMBB	Em-BOT	Er-h-BOT	EXBB	ERRB	EMQP	EMCA	EMDH	Er-HOO	ERDH	ERSDH	ERPE	ERPA	ERTS	ERDM	ERDW	ERMK	ESTL	Er-MOZ	CBS	E	PS	SH
EMDP																																		
EMPN	0.3830																																	
EMKZ	0.4276	0.0483																																
EMHK	0.3463	0.0703	0.1091																															
EMDB	0.5019	0.2949	0.2920	0.1534																														
EMJB	0.3074	0.1536	0.1716	0.0902	0.1519																													
EMKM	0.3117	0.1573	0.1707	0.0849	0.1356	0.0235																												
EMMP	0.3562	0.2057	0.2163	0.1440	0.1843	0.0492	0.0436																											
EMVM	0.5343	0.4029	0.3379	0.3571	0.4682	0.2832	0.2906	0.3683																										
EMSJ	0.3354	0.2017	0.2058	0.1329	0.1636	0.0318	0.0155	0.0266	0.3249																									
Em-MUI	0.4041	0.2343	0.2099	0.1701	0.2231	0.0867	0.0810	0.1146	0.2762	0.0958																								
EMBB	0.3976	0.1913	0.1988	0.1085	0.1237	0.0556	0.0322	0.0168	0.3752	0.0342	0.1124																							
Em-BOT	0.4396	0.2741	0.2608	0.2132	0.3034	0.1514	0.1656	0.1898	0.2798	0.1748	0.1702	0.1845																						
Er-h-BOT	0.5670	0.4404	0.4374	0.3825	0.4385	0.2762	0.2790	0.3018	0.5108	0.2928	0.3385	0.3186	0.4014																					
EXBB	0.5807	0.4601	0.4453	0.4059	0.4847	0.3061	0.3259	0.3622	0.4406	0.3434	0.3545	0.3785	0.2991	0.1407																				
ERRB	0.5638	0.4249	0.4422	0.3527	0.4328	0.2667	0.2687	0.3394	0.4841	0.3105	0.2455	0.3387	0.3923	0.5194	0.5308																			
EMQP	0.3546	0.2043	0.2050	0.1170	0.1269	0.0285	0.0242	0.0404	0.3227	0.0143	0.0875	0.0443	0.2012	0.2761	0.3369	0.2876																		
EMCA	0.4002	0.2249	0.2020	0.1608	0.1681	0.0583	0.0628	0.0695	0.3237	0.0538	0.0874	0.0725	0.1988	0.2909	0.3381	0.3006	0.0345																	
EMDH	0.5406	0.2851	0.2460	0.2259	0.3135	0.1762	0.1683	0.2192	0.4033	0.1883	0.1316	0.2051	0.2903	0.4154	0.4383	0.3911	0.1732	0.1675																
Er-HOO	0.4493	0.2429	0.2356	0.1662	0.2103	0.0965	0.1076	0.1164	0.4186	0.0915	0.1508	0.1180	0.2374	0.3390	0.3853	0.3775	0.0843	0.1042	0.0792															
ERDH	0.5290	0.3014	0.2670	0.2529	0.3399	0.1562	0.1651	0.1982	0.4089	0.1549	0.1448	0.2019	0.2753	0.3714	0.3961	0.4062	0.1689	0.1587	0.0381	0.0758														
ERSDH	0.4638	0.3415	0.3362	0.2486	0.3875	0.1372	0.1450	0.1879	0.3682	0.1777	0.1491	0.2332	0.2716	0.4003	0.4133	0.4112	0.1481	0.1463	0.2476	0.1916	0.2379													
ERPE	0.7251	0.6599	0.6542	0.6070	0.6990	0.5085	0.5273	0.5279	0.6628	0.5470	0.5302	0.5823	0.5754	0.6415	0.6317	0.6749	0.5376	0.5143	0.6354	0.5728	0.6118	0.4950												
ERPA	0.7272	0.6229	0.5965	0.5681	0.6778	0.4835	0.5080	0.5251	0.5393	0.5204	0.4928	0.5589	0.4983	0.6248	0.5716	0.6648	0.5068	0.4790	0.5684	0.5417	0.5557	0.5125	0.6406											
ERTS	0.6613	0.5611	0.5263	0.5129	0.6303	0.4328	0.4527	0.4757	0.4619	0.4719	0.4365	0.5085	0.4547	0.5486	0.4945	0.6222	0.4590	0.4337	0.4806	0.4714	0.4693	0.3922	0.5474	0.1454										
ERDM	0.5248	0.4210	0.4010	0.3741	0.5102	0.2991	0.3175	0.3370	0.3448	0.3394	0.3137	0.3722	0.3097	0.4719	0.4213	0.4668	0.3350	0.3099	0.3721	0.3542	0.3678	0.2063	0.4437	0.1981	0.1363									
ERDW	0.5317	0.4481	0.4494	0.4107	0.5346	0.3276	0.3559	0.3676	0.3986	0.3728	0.3365	0.4088	0.3754	0.4957	0.4707	0.4968	0.3586	0.3463	0.4238	0.3931	0.4213	0.2152	0.3714	0.3355	0.2507	0.1661								
ERMK	0.5855	0.4951	0.4989	0.4329	0.5814	0.3609	0.3697	0.3936	0.4738	0.4073	0.3682	0.4262	0.3819	0.5710	0.5421	0.5454	0.4128	0.4058	0.5011	0.4744	0.4864	0.3443	0.5569	0.5973	0.5212	0.3596	0.3259							
ESTL	0.5936	0.4939	0.4758	0.4256	0.5823	0.3226	0.3495	0.3869	0.4061	0.3824	0.3198	0.4278	0.3998	0.5666	0.5433	0.5532	0.3826	0.3963	0.4437	0.4337	0.4299	0.3285	0.6313	0.6311	0.5146	0.3837	0.3419	0.3623						
Er-MOZ	0.5619	0.4794	0.4642	0.4264	0.5504	0.3216	0.3491	0.3884	0.4111	0.3760	0.3223	0.4235	0.4006	0.5449	0.5228	0.5243	0.3839	0.3913	0.4509	0.4378	0.4242	0.3361	0.6160	0.6310	0.5448	0.4309	0.4091	0.4030	0.1075					
CBS	0.5704	0.4720	0.4677	0.4237	0.5629	0.3324	0.3597	0.3858	0.4364	0.3783	0.3330	0.4121	0.3912	0.5435	0.5268	0.5334	0.3887	0.3968	0.4555	0.4352	0.4387	0.3485	0.5631	0.5839	0.4948	0.3666	0.3030	0.3539	0.2572	0.3240				
E	0.5088	0.4171	0.4162	0.3753	0.5107	0.2961	0.3180	0.3476	0.3858	0.3405	0.2952	0.3718	0.3584	0.5008	0.4862	0.4693	0.3481	0.3566	0.4041	0.3937	0.3969	0.2554	0.4894	0.5205	0.4334	0.2975	0.2613	0.3065	0.1792	0.2506	0.0460			
PS	0.5081	0.4310	0.4194	0.3839	0.5192	0.3003	0.3244	0.3492	0.3791	0.3446	0.2941	0.3795	0.3645	0.5051	0.4884	0.4856	0.3524	0.3628	0.4066	0.3968	0.3967	0.2776	0.5037	0.5338	0.4289	0.3149	0.2680	0.3197	0.1358	0.2289	0.0648	0.0238		
SH	0.5338	0.4350	0.4283	0.3966	0.5352	0.3210	0.3442	0.3688	0.3995	0.3681	0.3204	0.3890	0.3820	0.5235	0.5093	0.5094	0.3766	0.3827	0.4120	0.4107	0.4101	0.2983	0.5141	0.5458	0.4459	0.3284	0.2703	0.3142	0.2147	0.2943	0.0523	0.0302	0.0368	

B)

Sample	EMDP	EMPN	EMKZ	EMHK	EMDB	EMJB	EMKM	EMMP	EMVM	EMSJ	Em-MUI	EMBB	Em-BOT	Er-h-BOT	EXBB	ERRB	EMQP	EMCA	EMDH	Er-HOO	ERDH	ERSDH	ERPE	ERPA	ERTS	ERDM	ERDW	ERMK	ESTL	Er-MOZ	CBS	E	PS	SH
EMDP																																		
EMPN	0.2089																																	
EMKZ	0.2697	0.0177																																
EMHK	0.2487	0.0377	0.0681																															
EMDB	0.3233	0.1877	0.1709	0.1247																														
EMJB	0.3421	0.1381	0.2087	0.0838	0.1578																													
EMKM	0.3131	0.1429	0.1960	0.0922	0.1372	0.0470																												
EMMP	0.3431	0.1567	0.2629	0.1112	0.1584	0.0437	0.0563																											
EMVM	0.4052	0.3607	0.2644	0.3995	0.2942	0.3068	0.2341	0.3397																										
EMSJ	0.3206	0.1944	0.2691	0.1593	0.1335	0.0395	0.0168	0.0225	0.2643																									
Em-MUI	0.4404	0.2650	0.2799	0.1800	0.1606	0.0953	0.0650	0.0446	0.2627	0.0297																								
EMBB	0.3406	0.1261	0.1848	0.0595	0.0980	0.0689	0.0310	0.0080	0.3306	0.0218	0.0404																							
Em-BOT	0.4824	0.2464	0.2710	0.1700	0.2526	0.1685	0.1148	0.0888	0.2181	0.0951	0.0931	0.0561																						
Er-h-BOT	0.4865	0.3493	0.3680	0.3554	0.2919	0.2731	0.2876	0.2793	0.5405	0.2734	0.4368	0.2796	0.5691																					
EXBB	0.6301	0.5393	0.5125	0.5464	0.5191	0.4905	0.5316	0.5290	0.4201	0.5165	0.5766	0.5211	0.2964	0.0275																				
ERRB	0.4046	0.3435	0.4157	0.3004	0.3042	0.2850	0.2855	0.3166	0.3942	0.3057	0.2131	0.2980	0.4341	0.4985	0.6413																			
EMQP	0.3234	0.1869	0.2504	0.1260	0.1094	0.0345	0.0381	0.0416	0.2476	0.0272	0.0316	0.0313	0.1647	0.2490	0.4896	0.2583																		
EMCA	0.4497	0.2226	0.2512	0.1701	0.1359	0.0714	0.1003	0.0683	0.3261	0.0631	0.0748	0.0631	0.1696	0.2685	0.5143	0.3474	0.0330																	
EMDH	0.5171	0.2376	0.1887	0.1776	0.2039	0.1624	0.1404	0.1421	0.3558	0.1129	0.0786	0.1045	0.1922	0.4006	0.5429	0.3212	0.1133	0.1130																
Er-HOO	0.4205	0.1977	0.2196	0.1284	0.1416	0.0926	0.1077	0.0660	0.5341	0.0627	0.1303	0.0542	0.1930	0.2766	0.5171	0.3662	0.0684	0.0756	0.0144															
ERDH	0.5833	0.2884	0.2348	0.2764	0.3055	0.1848	0.1776	0.1617	0.4484	0.1053	0.1313	0.1623	0.2494	0.3158	0.4645	0.4223	0.1638	0.1384	0.0134	0.0335														
ERSDH	0.4587	0.4006	0.4047	0.3589	0.4646	0.2512	0.2698	0.2948	0.4542	0.2717	0.2750	0.3162	0.4965	0.5225	0.7096	0.4628	0.2351	0.2458	0.2704	0.2703	0.2800													
ERPE	0.6942	0.8278	0.8877	0.8490	0.9266	0.7777	0.8212	0.7056	0.7521	0.7906	0.8282	0.8282	0.8939	0.6276	0.6819	0.6088	0.7527	0.7643	0.8776	0.7831	0.8335	0.3808												
ERPA	0.8680	0.7946	0.6199	0.7510	0.9255	0.8353	0.8287	0.8400	0.4534	0.8085	0.7388	0.8388	0.6691	0.7338	0.5769	0.7663	0.7706	0.7598	0.5607	0.7087	0.5586	0.4872	0.4616											
ERTS	0.7416	0.7629	0.5965	0.7732	0.9461	0.8123	0.8258	0.7867	0.4071	0.7770	0.7400	0.8165	0.6562	0.5802	0.4410	0.8284	0.7555	0.7233	0.4821	0.6236	0.4848	0.4683	0.3523	0.0482										
ERDM	0.6487	0.5711	0.4996	0.5630	0.8461	0.6154	0.6347	0.5391	0.3581	0.6004	0.6307	0.5928	0.4639	0.7080	0.5816	0.5996	0.6316	0.6152	0.4563	0.5586	0.4773	0.3439	0.3659	0.0908	0.0622									
ERDW	0.5935	0.5751	0.6161	0.6387	0.9007	0.6326	0.7016	0.7177	0.4267	0.7026	0.6626	0.7926	0.7075	0.8267	0.7413	0.6533	0.6680	0.7364	0.6309	0.7479	0.6787	0.2775	0.2687	0.2624	0.2073	0.2195								
ERMK	0.5289	0.5060	0.5229	0.4962	0.6829	0.5706	0.4784	0.4190	0.4337	0.5192	0.4636	0.4305	0.4013	0.7783	0.7161	0.5697	0.5656	0.5752	0.5574	0.6741	0.6064	0.3628	0.4104	0.6065	0.5671	0.3840	0.3303							
ESTL	0.4699	0.5598	0.4558	0.5154	0.6463	0.4818	0.4923	0.4628	0.3230	0.4911	0.4535	0.4862	0.5036	0.7179	0.7247	0.5472	0.4849	0.6343	0.4049	0.4994	0.4600	0.3805	0.4426	0.4614	0.4421	0.3840	0.3600	0.2266						
Er-MOZ	0.4582	0.5047	0.4718	0.5322	0.6025	0.4003	0.4552	0.3994	0.2669	0.3967	0.3698	0.4506	0.4478	0.6661	0.6827	0.4840	0.4391	0.5099	0.3713	0.4870	0.3941	0.2992	0.5305	0.6895	0.6382	0.5623	0.4259	0.2977	0.0560					
CBS	0.4971	0.4873	0.4992	0.4783	0.6212	0.4211	0.4612	0.4388	0.3952	0.4828	0.3972	0.4598	0.4943	0.6724	0.6790	0.4820	0.5113	0.5920	0.4342	0.5853	0.4609	0.4171	0.3885	0.4698	0.4560	0.3860	0.2904	0.2369	0.1494	0.2153				
E	0.4812	0.4750	0.4967	0.4801	0.6752	0.4743	0.5146	0.4372	0.3837	0.5154	0.4419	0.4885	0.5192	0.7131	0.7373	0.4414	0.5484	0.6330	0.4413	0.5958	0.5176	0.3427	0.3875	0.4640	0.4465	0.2424	0.2950	0.2214	0.1194	0.1903	0.0259			
PS	0.4267	0.5172	0.4914	0.5142	0.6165	0.4781	0.5071	0.4367	0.3536	0.4872	0.4266	0.5019	0.5339	0.6904	0.7070	0.4835	0.5474	0.6210	0.4452	0.5883	0.5014	0.3910	0.3713	0.4017	0.3714	0.2751	0.3008	0.2621	0.0856	0.1658	0.0508	0.0174		
SH	0.4689	0.4393	0.4642	0.4484	0.6346	0.4308	0.4680	0.3976	0.3727	0.4652	0.4424	0.4088	0.4852	0.6908	0.7120	0.5076	0.5021	0.5818	0.3939	0.5320	0.4780	0.2756	0.3709	0.4636	0.4230	0.3102	0.2638	0.2136	0.1151	0.1998	0.0269	0.0220	0.0193	

Table S5 - Number of records and performance of species distribution models inferred with cross-validation (CV) and the final predictive models.

**Species**	n	AUC (CV)	Sensitivity (CV)	AUC (Final)	Sensitivity (Final)
Ecklonia radiata	36639	0.886	0.968	0.907	0.973
Ecklonia maxima	466	0.834	0.923	0.919	0.977

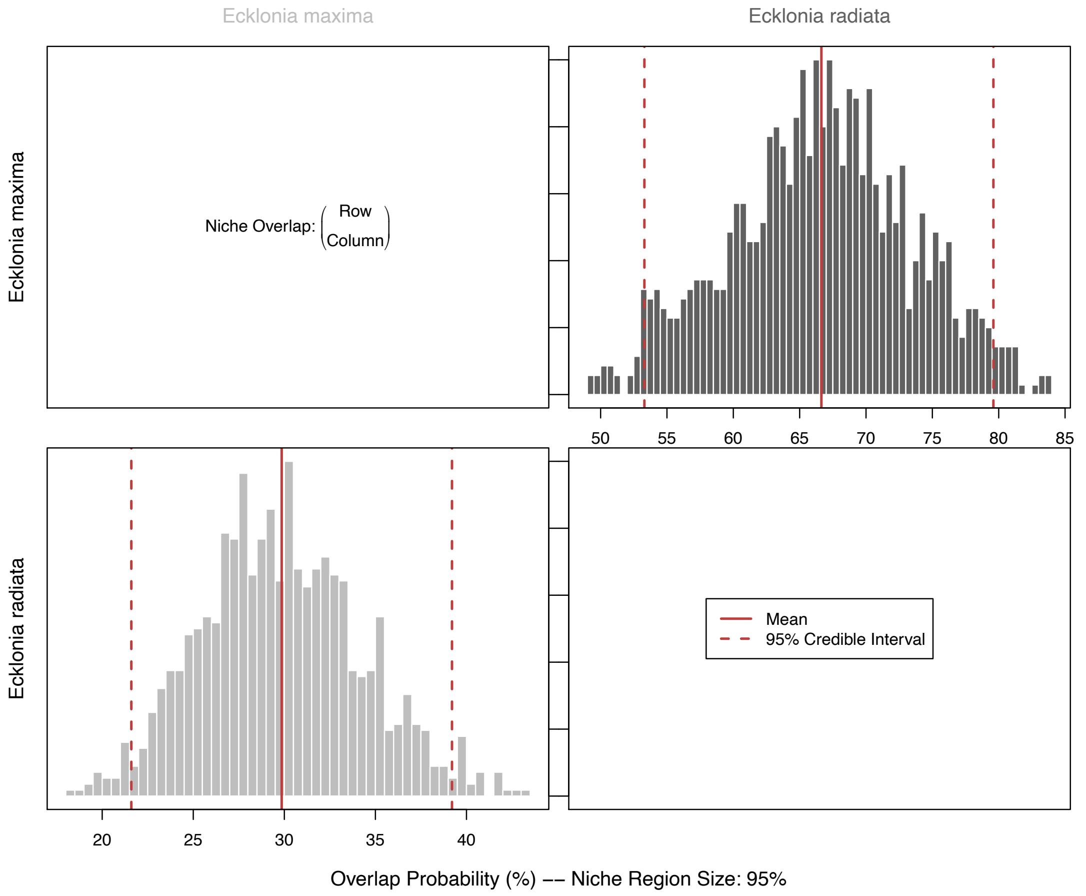

Figure S1. Posterior distribution of the probabilistic niche overlap between *Ecklonia radiata* and *Ecklonia maxima.* The panels indicate the probability of an individual from the species of rows being found within the ecological niche of the species of columns.

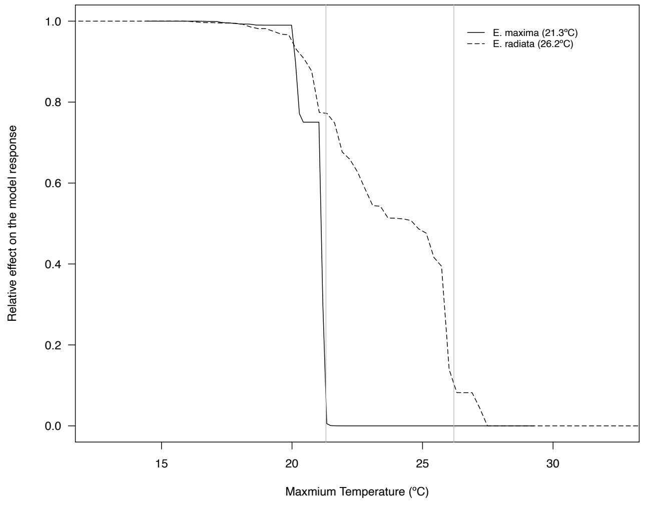

Figure S2. Thermal tolerance limits inferred for *Ecklonia radiata* and *Ecklonia maxima* with Species Distribution Modelling.

Table S6. Niche similarity between *Ecklonia radiata* and *Ecklonia maxima*.

D / I	*Ecklonia radiata*	*Ecklonia maxima*
Ecklonia radiata	-	0.07 / 0.17
Ecklonia maxima	0.07 / 0.17	-

Table S7. Niche similarity tests (p-values) performed between *Ecklonia radiata* and *Ecklonia maxima*.

D / I	*Ecklonia radiata*	*Ecklonia maxima*
Ecklonia radiata	-	n.s / n.s
Ecklonia maxima	n.s / n.s	-

Table S8. Probabilistic niche overlap between hybrids found in genetic analyses and *Ecklonia radiata* and *Ecklonia maxima.* Values indicate the probability of a hybrid individual being found within the ecological niche of *Ecklonia radiata* or *Ecklonia maxima*. Bold values depict the higher probability of comparisons.

Site	*Ecklonia radiata*	*Ecklonia maxima*
ERSDH	27.00	**80.98**
EXBB	**86.73**	61.82
Er-h-BOT	**87.00**	61.61
EMVM	**78.37**	66.44
ERRB	**78.43**	66.91
